# Identification of Crucial Gene Modules Related to the Efficiency of Anti-PD-1/PD-L1 Therapy and Comprehensive Analyses of a Novel Signature Based on These Modules

**DOI:** 10.3389/fgene.2022.893380

**Published:** 2022-07-22

**Authors:** Wei Wang, Dong Dong, Liang Chen, Heng Wang, Bo Bi, Tianyi Liu

**Affiliations:** ^1^ Department of Plastic and Aesthetic Surgery, Huadong Hospital, Fudan University, Shanghai, China; ^2^ Shanghai Medical College of Fudan University, Shanghai, China; ^3^ Department of Cosmetic Surgery, Shanghai Changning Maternity and Infant Health Hospital, Shanghai, China

**Keywords:** melanoma, TCGA, signature, immunotherapy, tumor microenvironment

## Abstract

Biomarker development for clinical checkpoint inhibition is still in its early stages. It is critical to determine the cause of the lack of a long-term response in patients after immune checkpoint blockade (ICB) treatment and to develop composite biomarkers or signatures to improve personalized approaches. Three modules that were significantly correlated with the immunotherapeutic response were identified. Stimulatory pathways of cellular immunity, extracellular matrix formation-related pathways, and ATP metabolism-related pathways were enriched. Two distinct transcriptional subtypes were determined. Tumor microenvironment (TME) characteristics were highly correlated with “hot” and “cold” tumors. The ICB score was significantly correlated with clinical characteristics including age, Breslow depth, Clerk level, AJCC stage, and T stage. Meanwhile, a low ICB score is characterized by increased activation of immunity, a higher level of immune infiltration, and immune molecule expression. The ICB score showed a robust ability to predict melanoma prognosis in the discovery, internal validation, and external validation cohorts. In addition, a low ICB score was linked to a higher CR/PR rate in the immunotherapeutic cohort. The ICB score could reflect the pre-existing immune features and the expression pattern of “Cold” versus “Hot” tumors in melanoma patients. Thus, it has the potential to serve as a reliable predictor of melanoma prognosis and response to ICB therapy.

## Introduction

Despite having a lower prevalence than other cutaneous malignancies, skin melanoma is one of the most aggressive cancers. Until recently, melanoma accounted for only 4% of all dermatological cancers, yet it was responsible for 80% of skin cancer-related mortality (Miller and Mihm, 2006). Non-invasive melanoma has a favorable surgical prognosis; nevertheless, metastatic melanoma has long lacked curative therapy options (Barrios et al., 2020). Although immune checkpoint blockade (ICB), represented by anti-PD-1/L1, has changed cancer therapy, only a small percentage of cancer patients (10–30%) have a long-term clinical response (Topalian et al., 2012) (Khalil et al., 2016). As a result, discovering the mechanisms of pre-existing and acquired immune resistance and developing new therapeutic techniques to prevent relapse are of great interest. It is also critical to determine the cause of the lack of long-term response in patients after ICB treatment and to develop composite biomarkers or signatures to improve personalized approaches.

Biomarker development for clinical checkpoint inhibition is still in its early stages of development. The tumor mutation burden and PD-L1 expression are the only biomarkers used in some cases. Recent research has found that PD-L1 expression measured by immunohistochemistry (IHC) and tumor mutation burden (TMB) measured by whole exome sequencing (WES) are not reliable predictors of ICB response in a variety of tumor types (Merino et al., 2020) (McGrail, Pilié, Rashid, Voorwerk, Slagter, Kok, et al.). Pre-therapy expression analyses on pre-existent immune features in responders and expression patterns of “Cold” versus “Hot” tumors based on prior immunotherapy experience could predict immunotherapy response, according to mounting evidence (Riaz et al., 2017). Recently, several gene signatures have been established to predict prognosis and immunotherapeutic responses. These signatures, which included a variety of genes acquired from RT-PCR or RNA-seq data, exhibited good predictive powers. Still, none of them could be employed in clinical practice because of the insufficient robustness or lack of further validation (Xiong et al., 2020). Yan et al. developed a 9-gene signature correlated with effector T cell infiltration to predict immune checkpoint therapy response (Yan et al., 2021). However, the predictive ability of signature has not been clarified. A signature-based on immune infiltrating related genes has been constructed by Zhang et al. (Zhang et al., 2020). It showed a satisfactory diagnostic performance with an AUC value of 0.70–0.72 in the discovery cohort, while the AUC value in the test cohort was 0.60–0.67. In addition to the immune infiltration, other inherent characteristics of tumors have played a crucial role in affecting the immune therapy response, such as the immunogenicity of tumor cells, extracellular matrix components, and metabolic imbalance reprogramming of tumor cells. WGCNA is a classic data reduction and unsupervised classification method, which has been employed in thousands of transcriptional data analyses (Langfelder and Horvath, 2008). There is a consensus that the expression pattern of the patient’s transcriptome before treatment primarily affects the efficacy of immune checkpoint therapy. However, as its complex and dynamic nature, our understanding of the expression feature relating to the efficiency of immunotherapy remains incomplete (Chen and Mellman, 2017).

For the first time, we applied the WGCNA method to mRNA sequencing data of patients receiving anti-PD-1 therapy to discern the hub gene modules directly associated with therapeutic response. We analyzed six melanoma datasets from The Cancer Genome Atlas (TCGA) and Gene Expression Omnibus (GEO) database in the present study. First, we identified three key modules and the corresponding biological processes associated with the efficacy of PD-1 inhibition therapy based on the ICB cohort (GSE91061). Then we revealed two distinct transcriptional subtypes based on key modules and discovered that the tumor microenvironment (TME) under them was highly consistent with the “hot” and “cold” tumor. Furthermore, we developed a robust signature (ICB score) based on key modules that were significantly related to immunotherapeutic efficiency. Predictive ability was verified using an internal validation cohort and two independent GEO cohorts. Our study demonstrated that the ICB score is a robust biomarker of prognosis and immunotherapeutic response.

## Methods and Materials

### Data Acquisition and Preprocessing

The gene expression profile and corresponding clinical information of patients with melanoma were obtained from the publicly available databases TCGA and GEO. Five eligible cohorts (TCGA-SKCM, GSE65904, GSE22513, GSE91061, and IMvigor210) were included in this study. Among them, GSE91061 and IMvigor210 were the immunotherapeutic cohorts. TCGA RNA sequencing data (TCGA-SKCM, FPKM format) were obtained from the UCSC Xena database (https://gdc.xenahubs.net/download/TCGA-SKCM.htseq_fpkm.tsv.gz). For GSE91601, we downloaded the raw “CEL” files and used a robust multiarray averaging method with “simpleaffy” and “affy” packages to perform background adjustment and quantile normalization. For sequencing data of other GEO cohorts, we directly obtained the normalized matrix files from the GEO database. We analyzed each dataset separately in this study rather than merging them into a larger cohort. The somatic mutation and SCNV data of TCGA-SKCM were also downloaded from the TCGA database. Data processing was performed using the R (version 4.0.5) and R Bioconductor packages.

### Co-Expression Modules Construction

Weighted Gene Co-expression Network Analysis (WGCNA) is a method for dividing the whole RNA expression profile into co-expression gene modules and investigating the link between modules and phenotypic features (Zhang and Horvath, 2005). The standard deviation of a single gene greater than 0.5 was set as the inclusion criterion to perform WGCNA using the expression data of the TCGA cohort in our investigation. The scale independence and average connection degree of the network with various power values were investigated (ranging from 1 to 20). When the scale independence was greater than 0.9 and connectedness was higher, a suitable power value was found. Genes were then grouped into distinct gene modules based on topological overlap matrix (TOM)-based dissimilarity. The main module was determined to have the strongest association with immunotherapy response.

### Unsupervised Consensus Molecular Clustering Based on Genes in the Key Module

An unsupervised clustering algorithm was used to classify patients for further research and uncover different expression patterns based on genes in critical modules. The consensus clustering algorithm determines the number of clusters and their stability (Yu et al., 2012). We used the ConsensuClusterPlus package to perform the above steps and conducted 1,000 repetitions to guarantee the stability of the classification (Wilkerson and Hayes, 2010).

### Pathway Enrichment Analysis

The clusterProfiler R package was used to perform functional annotation for genes in modules that were substantially (*p* < 0.05) connected with the response to nivolumab (anti-PD-1 drug) treatment to study the pathways enriched in important modules. We used the “GSVA” R package to perform GSVA enrichment analysis to determine differences in biological processes across diverse transcriptional subtypes defined by key module genes. In a non-parametric and unsupervised technique, GSVA is often used to estimate the variance in biological route and process activity in expression dataset samples (Hänzelmann et al., 2013). For GSVA analysis, the gene sets “c2. cp.kegg.v6.2. symbols” were retrieved from the MSigDB database. Statistical significance was defined as an adjusted P-value of less than 0.05. The TCGA-SKCM cohort was subjected to gene set enrichment analysis (GSEA) using GSEA software (version 4.1.0) to determine the biological process differences between the high-risk and low-risk groups. For GSEA analysis, statistical significance was defined as a False Discovery Rate (FDR) of less than 0.05. We used biological processes or signatures yielded by previous research\ and ssGSEA methods to characterize the stromal activation and immune program of melanoma patients (Mariathasan et al., 2018) (Şenbabaoğl u et al., 2016). The biological processes or signatures which were characterized in our study included angiogenesis signature, antigen processing machinery signature, CD8 T effector cell signature, EMT signature, FGFR3-related signature, cell cycle signature, and repair of DNA damage signature (BER, FA, HR, MMR, NHEJ, and NER).

### Immune Infiltration and Immune Status Estimation

We adopted the Timer (Li et al., 2016), CIBERSORT (Newman et al., 2015), CIBERSORT-ABS, QUANTISEQ (Finotello et al., 2019), MCPCOUNTER (22), Xcell (Aran et al., 2017), and EPIC(24) algorithms to compare the cellular components between the different transcriptional subtypes and the high and low ICB score groups, respectively. In addition, we quantified the relative abundance of each cell infiltrate in the TME of SKCM using single-sample gene set enrichment analysis (ssGSEA). The gene set for identifying each TME infiltrating immune cell type was derived from Charoentong’s work, which included activated CD8 T cells, activated dendritic cells, macrophages, natural killer T cells, regulatory T cells, and other human immune cell subtypes (Charoentong et al., 2017) (Barbie et al., 2009).

### Construction of the ICB Score

Least Absolute Shrinkage and Selection Operator regression (LASSO) is a penalized regression that can generate risk models by screening variables from high-dimensional data (Gui and Li, 2005). Patients in the TCGA cohort were separated into two groups in a 7:3 ratio in our study: discovery (n = 322) and internal validation (n = 136). LASSO regression was used in the discovery cohort to identify the most valuable genes with predictive potential for SKCM. Using the minimal criterion, ten-time cross-validation was used to determine the optimal value of the tuning parameter (λ). A gene signature was created using multivariate Cox regression analysis. A risk score formula was created based on the signature that we created.

$$ICB\;score\;=\;\underset{i}{\sum }Coefficient\;of\;the\;gene\;\left(i\right)\times Expreesion\;of\;gene\;\left(i\right)$$

*The coefficient of the gene (i)* is the regression *coefficient of the gene* in the LASSO-Cox regression model, and the expression of the gene (i) is the expression value of gene for each patient. The best cutoff value was established using the surv-cutpoint function from the ‘survival’ package, and patients were separated into high- and low-risk groups.

### Comparison of Genomic Alterations in Different Melanoma Subtypes

GDC was used to obtain TCGA-SKCM mutation data (VarScan2). The analysis eliminated genes with mutation rates of less than 2.5 percent. GDC was used to acquire the TCGA-SKCM SCNV data. GISTIC software (version 2.0) was used to examine the GISTIC score and gene copy number amplification and deletion data for each sample. Each melanoma sample’s fraction of genome gained or lost (FGG, FGL) and genome altered (FGA) value was calculated (Moore et al., 2012). We compared the FGG, FGL, and FGA values between genders, ages, TNM stages, and ICB scores. The R program RCircos was used to visualize the location of the gene on the chromosome.

### Quantify the Immune Response Predictor: Immunophenoscore, TIDE, ESTIMATE

The immunophenotype score (IPS) is a better predictor of the response to anti-CTLA-4 and anti-PD-1 therapies because it quantifies tumor immunogenicity and characterizes intratumoral immune landscapes and cancer antigenomes (Becht et al., 2016). A panel of immune-related genes from the four groups of MHC-related molecules (MHC), checkpoints or immunomodulators (CP), effector cells (EC), and suppressor cells (SC) were used to establish the scoring methodology (SC). The weighted averaged Z-score was determined by averaging the sample-wise Z-scores of the four classes within each category. The total averaged Z-score was calculated as the IPS. Jiang et al. presented the Tumor Immune Dysfunction and Exclusion (TIDE) algorithm, which was used to represent several tumor immune evasion mechanisms (Jiang et al., 2018), including tumor infiltrating cytotoxic T lymphocytes (CTLs) dysfunction and CTL exclusion by immunosuppressive substances. A higher TIDE score indicated that tumor cells were more likely to trigger immunological escape, implying a lower ICI response rate. The Estimation of Stromal and Immune Cells in Malignant Tumors using Expression Data (ESTIMATE) algorithm (Yoshihara et al., 2013) uses the unique qualities of transcriptional patterns to estimate tumor cellularity and purity. We derived immune and stromal scores using the ESTIMATE method to assess the levels of infiltrating immune and stromal cells, which were used to infer tumor purity.

### Statistical Methods

R-4.1.0) was used to perform statistical analyses in this study. The correlation coefficients were calculated using Spearman’s sand distant correlation analyses in this study. Student’s t-tests were used to quantify statistical significance for normally distributed variables, whereas the Wilcoxon rank-sum test was used to examine abnormally distributed variables. Kruskal-Wallis tests and one-way analysis of variance were used as non-parametric and parametric procedures for comparisons of more than two groups, respectively (Hazra and Gogtay, 2016). In this work, the survival curve was plotted by Kaplan-Meier (KM) techniques and evaluated by Log-rank methods using the R package “Survminer” and “Survival”, respectively (0.4.6). The relationship between ICB score and clinical variables and prognosis was investigated using the Cox proportional hazards model. The surv-cutpoint function from the ‘survival’ package was used to stratify samples into high and low ICB score subgroups. The prognostic classification performance of the ICB score model was evaluated using the receiver operating characteristic (ROC) curve, and the area under the curve (AUC) was determined using the ‘timeROC’ package (0.3). The Benjamini-Hochberg technique and Bonferroni method were used to control the false discovery rate (FDR) for multiple hypothesis testing (Love et al., 2014). All comparisons were two-sided, with an alpha level of 0.05.

### Nomogram Construction

The nomogram was constructed using the R package “rms”. Calibration curves were used to test the consistency between the projected and actual survival outcomes. Time-dependent ROC curves were used to assess the prediction accuracy of the nomogram, gene risk model, and clinicopathologic variables.

## Results

### Identification of Key Modules and Biological Processes in the Immunotherapeutic Cohort

**FIGURE 1:**
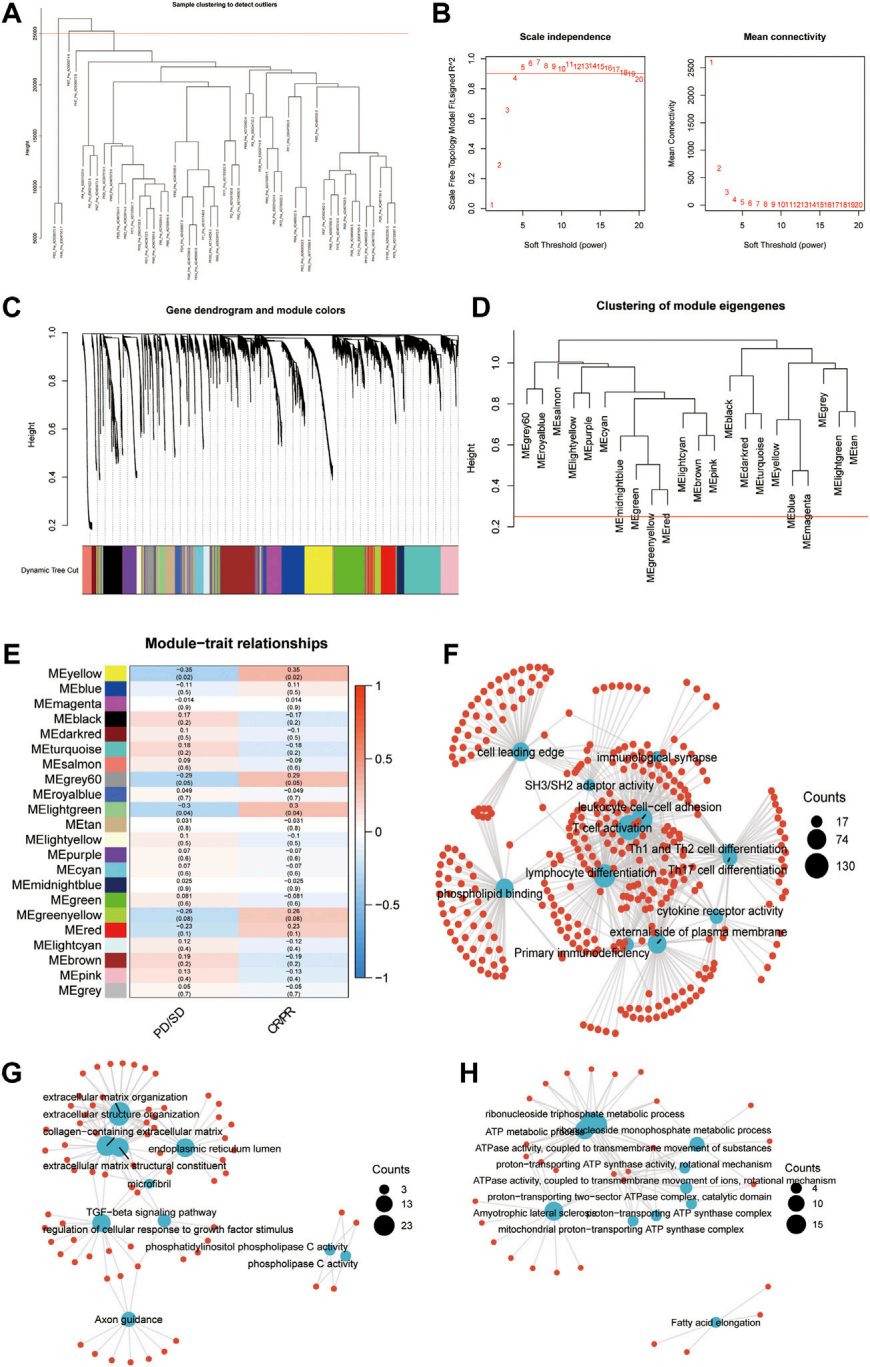
Key modules and biological processes relating to the response to anti-PD-1 therapy. **(A)** The cluster dendrogram displays the relationship between samples in GSE91061. **(B)** Analysis of the scale-free fit index (left) and the mean connectivity (right) for various soft-thresholding power values. **(C)** Dendrogram of all DEGs clustered based on a dissimilarity measure (1-TOM) together with assigned module colors. **(D)** Clustering of module eigengenes. The red line indicates cut height = 0.25. **(E)** Correlation heatmap between eigengenes and response to Nivolumab therapy. Each cell contained the Spearman coefficient and P-value. PD/SD, progressed disease or stable disease. CR/PR, complete response or partial response. **(F–H)** Enrichment network showing the pathways enriched in the yellow **(F)**, lightgreen **(G)**, and grey60 **(H)** modules. The blue node represented pathways. The red dot represented molecules. The line represented the relationship between the pathway and the molecule.

The pre-therapy transcriptional profile can reflect prestored immune patterns in responders and expression imprints of “Cold” versus “Hot” tumors based on prior immunotherapy discovery (McGrail, Pilié, Rashid, Voorwerk, Slagter, Kok, et al.; Cristescu et al., 2018). Many previous studies evaluated the sensitivity and specificity of classic biomarkers, especially PD-L1/IHC and TMB. While high PD-L1 expression and TMB were associated with increased ORR, as recently described by Cristescu et al. (Racle et al., 2017), in the context of PD-1 inhibition, these biomarkers exhibited low sensitivity, limited specificity, and therefore limited accuracy in identifying responders to immune checkpoint blockade. According to a systematic evaluation conducted by Lu et al. (Lu et al., 2019), the sensitivity of PD-L1/IHC and TMB are 0.50 (0.48–0.53) and 0.57 (0.51–0.62), respectively. The specificity of PD-L1/IHC and TMB are 0.63 (0.62–0.65) and 0.70 (0.66–0.73). However, some authors have suggested that diagnostic tests used for patient selection should have AUCs of 0.80 or higher (Frati et al., 2012) (English et al., 2016). In addition, when combining these biomarkers, 10% of responders were both PD-L1 expression and TMB low, suggesting the existence of independent mechanisms of response to PD-L1 blockade. Considering the low sensitivity and limited specificity of classic biomarkers, including TMB and PD-L1, to identify responders to pembrolizumab caused by the multifactorial tumor-specific mechanisms of response (Banchereau et al., 2021; Litchf ield et al., 2021), we conducted WGCNA in an anti-PD-1 cohort (GSE91061) to identify the key modules significantly related to the response to immune checkpoint blockade therapy. After setting the soft-cut height as 0.25 and thresholding value as 4 (scale-free R2 = 0.9) (Figures 1A–D), we extracted 22 co-expression modules (Figure 1E). According to module significance analysis, the yellow module was most significantly correlated with response to anti-PD-1 immunotherapy, followed by the light green and grey60 modules (yellow R2 = 0.35, *p* = 0.02, light green R2 = 0.3, *p* = 0.04, grey60 R2 = 0.29, and *p* = 0.05).

KEGG and GO enrichment analyses were conducted to investigate the biological processes in the three key modules. Immune activation-related pathways, including leukocyte cell-cell adhesion, T cell activation, lymphocyte differentiation, and immunological synapse, were significantly enriched in the yellow module, which suggested that genes allocated into this module play a crucial role in the immune activation process (Figure 1F). Meanwhile, extracellular matrix formation-related pathways, such as collagen-containing extracellular matrix, extracellular matrix structural constituent, extracellular structure organization, extracellular matrix organization, and TGF−beta signaling pathway, showed an apparent association with the light green module (Figure 1G). The pathways enriched in the grey60 module were associated with ATP metabolism (Figure 1H). The above results indicated that in addition to the adaptive immune function, the extracellular environment and metabolic imbalance between tumor and immune cells significantly influence the response to immunotherapy, which is similar to previous studies (Jiang et al., 2019).

### Transcriptional Subtypes Identified by Hub Gene Modules Showed Distinct Immune Landscapes

**FIGURE 2:**
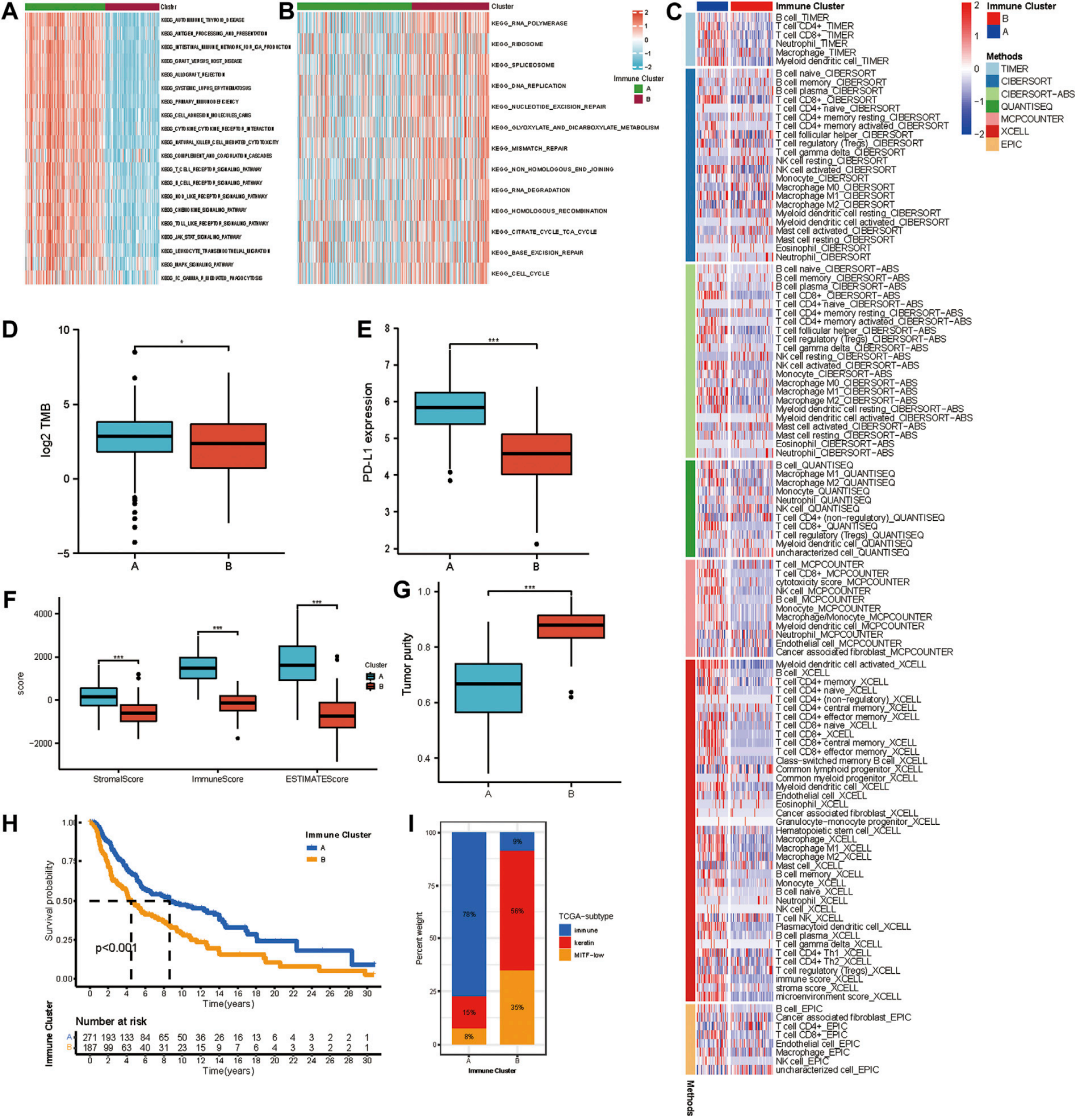
Different immune infiltrating landscape and biological characteristics in distinct transcriptional subtypes. **(A,B)** GSVA enrichment analysis showing the activation states of biological pathways in distinct transcriptional subtypes determined by genes in the yellow module. The heatmap was used to visualize these biological processes, and red represented activated pathways, and blue represented inhibited pathways. The activated KEGG pathways in Immune Cluster A (a); The inhibited KEGG pathways in Immune Cluster B (b). **(C)** Heatmap showing the cellular component of each melanoma sample estimated by seven algorithms in TCGA-SKCM cohort. **(D)** The difference in TMB between Immune Cluster A and B. **(E)** Difference in PD-L1 between Immune Cluster A and B. **(F)** Differences in the immune score, stromal score, and ESTIMATE score between Immune Cluster A and B. **(G)** Difference in tumor purity between Immune Cluster A and B. **(H)** Survival analyses for patients in Immune Cluster A and B in TCGA cohort using Kaplan-Meier method. **(I)** The distribution of TCGA subtypes in Immune Cluster A and B.

We selected the yellow, grey60, and light-green modules as the hub modules for further analysis. Based on the expression of genes in hub modules, the R package ConsensusClusterPlus was used to classify patients with qualitatively different transcriptional subtypes. Two distinct modification subtypes were eventually identified using unsupervised clustering, with 271 cases in subtype A and 187 cases in subtype B. These subtypes were termed Immune Cluster A-B. The distinction in the biological processes behind the two distinct immune-related transcriptional patterns was investigated using GSVA enrichment analysis. Figures 2A,B showed remarkably different pathways activated in immune clusters A and B. An FDR of less than 0.05 was considered statistically significant. Immune cluster A was enriched in cancer-immunity cycle stimulatory pathways, such as antigen processing and presentation, cytokine-cytokine receptor interaction, leukocyte transendothelial migration, and T/B cell receptor signaling pathway, and immune cluster B showed upregulated DNA damage repair processes such as nucleotide excision repair, mismatch repair, non-homologous end joining, and base excision repair. TIMER, CIBERSORT, CIBERSORT-ABS, QUANTISEQ, MCP counter, Xcell, and EPIC algorithms were used to create a heatmap of tumor-infiltrating immune cells. In immune cluster A, the majority of immunologically invading cells, notably CD8+ T lymphocytes, were identified in higher numbers (Figure 2C). We then evaluated the differences in the previously described biomarkers TMB (Figure 2D) and PD-L1 (Figure 2E) among these two clusters. Both showed a pronounced elevation in immune cluster A expression. We also used the ESTIMATE method to determine overall immune cell infiltration (immune score), stromal cell infiltration (stromal score), and tumor cell purity (tumor purity) for the two distinct subtypes. The results revealed that immune cluster A exhibited higher immune scores, stromal scores, and estimate scores than immune cluster B, while immune cluster B shared a higher tumor purity (Figures 2F,G). Consistent with previous studies, high levels of immune infiltration could serve as an independent biomarker for a favorable prognosis (Badalamenti et al., 2019) (Picard et al., 2020). Survival analysis showed that patients in immune cluster A shared a markedly prolonged OS compared to those in immune cluster B (Figure 2H). To study the characteristics of these distinct transcriptional patterns, we evaluated the differences and correlations between immune clusters and TCGA subtypes. Immune cluster A, which showed a more favorable survival, was enriched in the “immune” subtype but not in the “keratin” subtype and MIFF-low subtype (Figure 2I).

### Generation and Validation of ICB Score

The findings of the aforementioned clustering analysis revealed that SKCM patients could be appropriately categorized into “hot” and “cold” tumors, which were significantly related to the efficacy of immunotherapy. However, these findings were limited to a single patient group and could not reliably predict the pattern of hub gene expression in individual patients. Given the unique variability and complexity of hub gene expression patterns, we developed a scoring system based on these hub genes to quantify the expression patterns of individual patients with SKCM, which we call the ICB score. Based on the minimal partial likelihood deviation, LASSO regression was performed on 102 hub genes with prognostic significance in the discovery set, and ten biomarkers were preserved: STAT1, TNFSF10, CD40, CD40LG, TRIM22, PDCD1, GBP2, SEMA4D, ILR2B, and P2RY14. The ICB score was calculated as follows:

ICB score = (-0.3858 × expression of STAT1) + (0.7284 × expression of TNFSF10) + (-0.2900 × expression of CD40) + (0.2557 × expression of CD40LG) + (-0.4120 × expression of TRIM22) + (-0.2456 × expression of PDCD1) + (-0.4793 × expression of GBP2) + (-0.3629 × expression of SEMA4D) + (0.5350 × expression of IL2RB) + (-0.4539 × expression of P2RY14).

We explored the relationship of the expression patterns of signature genes with immune function and immune cell infiltrating levels. As shown in Supplementary Figure S2A, we can conclude that the expression levels of signature genes are all positively associated with the infiltrating levels of immune cells in CM tissues. The ICB signature established by LASSO methods included STAT1, TNFSF10, CD40, CD40LG, TRIM22, PDCD1, GBP2, SEMA4D, IL2RB, and P2RY14. Among them, 2 were classic immune co-stimulatory molecules (CD40 and CD40LG), 2 were signal transduction molecules promoting T cell activation (STAT1 and SEMA4D), 1 was immune checkpoint (PDCD1), 1 was immune-inflammatory molecule (TNFSF10), and 1 was immune-inflammatory molecular receptor (IL2RB). P2RY14 has been suggested to be a biomarker of tumor microenvironment immunomodulation and favorable prognosis in patients with head and neck cancer (Li et al., 2021). We focused on GBP2 and revealed its significant role in shaping the tumor microenvironment. GBP2, a GTP (guanylate-binding protein) superfamily member, plays a significant role in carcinoma. It was reported that GBP2 inhibits mitochondrial fission and cell invasion in several types of tumors (Ren et al., 2022). However, the function of GBP2 in melanoma has not been revealed. We observed that patients with higher GBP2 expression showed an obviously better outcome than patients with low GBP2 expression (*p* = 0.005, Supplementary Figure S2B). Besides, we further clarified the difference in the density of immune infiltrating cells between the low and high GBP2 groups. Significant differences in immune cell infiltrating levels, antigen presenting molecule expression, and immune checkpoint expression have been observed (Supplementary Figures S2C,D). From the above, we could speculate that GBP2 may promote the activation of various tumor killing immune cells and enhance the immune cells infiltrating within tumor tissues, thus enhancing the intratumoral antitumor immune response. CD8+ T cells are the primary effector immune cells in anti-tumor immunology. As shown in Supplementary Figure S3, significant positive correlations have been observed between the expression level of GBP2 and the infiltrating levels of CD8+ T cells. The result has been validated using various algorithms, including CIBERSORT, CIBERSORT-ABS, EPIC, MCPCOUNTER, QUANTISEQ, TIMER, and XCELL. To reveal the underlying mechanism of GBP2 in melanoma progression, we performed GSEA on sequencing data of TCGA-SKCM. The top 5 pathways enriched in high GBP2 expression were shown in Supplementary Figures S2E,F. The results suggested that GBP2 was significantly associated with chemokine, thereby affecting T cell infiltration. In addition, GBP2 also seems to be associated with the antigen processing pathway, which is vital to the activation of adaptive immunity. The multivariate COX regression analysis (Supplementary Figure S2G) suggested that GBP2 is an independent prognostic factor in melanoma, along with the AJCC T stage (*p* < 0.001) and N stage (*p* < 0.001). The above results provided new insight into the antitumor immunology of melanoma, although further validation using traditional experimental methods was needed.

**FIGURE 3:**
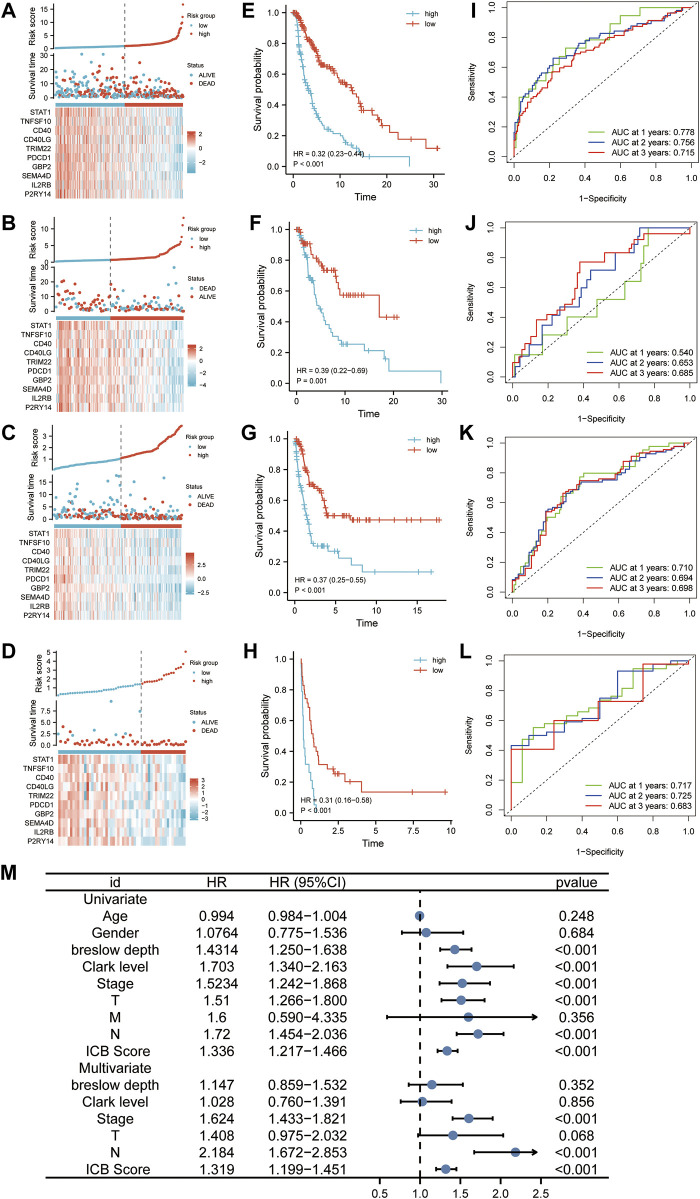
The predictability of ICB score in prognosis. **(A–D)** Ranked dot, scatter plots, and heatmap showing the ICB score distribution, patient survival status, and expression of genes generating the ICB score in TCGA discovery cohort **(A)**, TCGA internal validation cohort **(B)**, GSE65904 **(C)**, and GSE22153 **(D)**. **(E–H)** Survival curve showing the prognosis of patients with the low and high ICB scores in TCGA discovery cohort **(E)**, TCGA internal validation cohort **(F)**, GSE65904 **(G)**, and GSE22153 **(H)**. **(J-L)** Receiver operating characteristic (ROC) curve revealing the predictive ability of ICB score in TCGA discovery cohort **(I)**, TCGA internal validation cohort **(J)**, GSE65904 **(K)**, and GSE22153 **(L)** **(M)** Univariate and multivariate Cox regression analysis of the relationship between age, gender, Breslow depth, Clerk level, TMN stage, T stage, N stage, M stage, and the ICB score and prognosis.

According to the optimal cutoff value calculated by the “survminer” program, patients in the discovery, internal validation cohort (TCGA cohort), and two external validation datasets (GSE65904 and GSE22153) were divided into low and high ICB score groups. We used distribution plots, KM survival curves, and time-dependent ROC analyses in discovery (*n* = 322), internal (*n* = 136), and two external validation sets (GSE65094, *n* = 210; GSE22153, *n* = 54) to assess the signature’s predictive capacity (Figures 3A–D). Patients with high ICB scores had shorter survival times and higher mortality rates (Figure 3E) in the discovery set. The KM survival curve showed a markedly longer overall survival than patients with low scores (95%HR:0.23–0.44, *p* < 0.001). Consistent with the outcome in the discovery cohort, a lower survival rate and shorter survival time were observed in patients with higher ICB scores in the internal validation set (Figure 3F, 95%HR:0.22–0.69, *p* < 0.001), GSE65904 (Figure 3G, 95%HR:0.25–0.55, *p* < 0.001), and GSE22153 (Figure 3H, 95%HR:0.16–0.58, *p* < 0.001). At one, two, and 3 years, the prediction accuracies of this signature were 0.778, 0.756, and 0.715 in the discovery cohort,respectively (Figure 3I). In the internal validation dataset, the predictive accuracies of the ICB score were 0.540, 0.653, and 0.685 at 1, 2, and 3 years, respectively (Figure 3J). In addition, the area under the ROC curve of the ICB score for OS was 0.710, 0.694, and 0.698 at 1, 2, and 3 years, respectively, in GSE65904 (Figure 3K) and 0.717, 0.725, 0.683 at 1, 2, and 3 years, respectively, in GSE22153 (Figure 3L). According to the univariate findings, a lower ICB score was associated with unsatisfactory OS (hazard ratio [HR],1.336; 95% confidence interval [CI],1.217–1.466; *p* < 0.001). Higher Breslow depth, advanced Clerk level, TNM stage, T stage, N stage, and M stage are additional clinicopathological factors linked to poor survival. In multivariate analysis, ICB score (hazard ratio [HR]:1.319; 95% confidence interval [CI]:1.199–1.451; *p* < 0.001), along with TNM stage and N stage, remained independently linked with overall survival (Figure 3M).

### Clinical Characteristics and Functional Annotation

**FIGURE 4:**
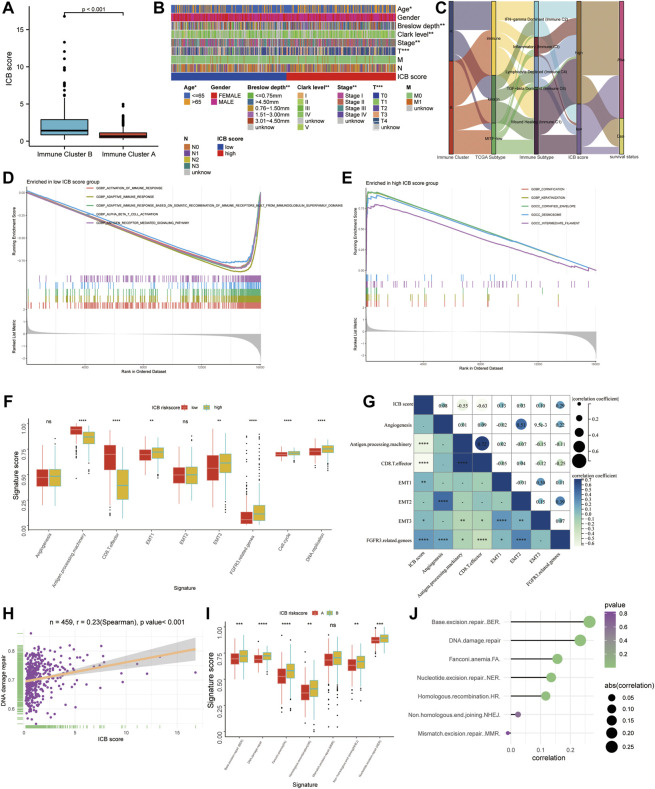
The functional annotation correlating to the ICB score. **(A)** The difference in the ICB scores between Immune Cluster A and B. **(B)** Heatmap on clinical characteristics revealing the differences in ages, Breslow depth, Clerk level, and TNM stage between the low and high ICB score groups. **(C)** Alluvial diagram showing the changes in the distribution of our Immune Cluster, TCGA Subtypes, Immune Subtypes, the ICB scores, and survival status. **(D–E)** GSEA analysis revealing the pathways enriched in the low ICB score group **(D)** and low ICB score group **(E)**. **(F)** Differences in specific pathways identified by Mariathasan et al., including immune activated, stromal activated, and DNA replication related pathways, between the low and high ICB score groups. **(G)** Correlation heatmap showing the association between the ICB score and pathways identified by Mariathasan et al. **(H)** scatter diagram showing the correlation between the ICB score and DNA damage repair. **(I)** Difference in DNA damage repair related pathways **(J)** Lollipop figure showing the correlation of the ICB score with DNA damage repair related pathways. ∗*p* < 0.05, ∗∗*p* < 0.01, ∗∗∗*p* < 0.001, ∗∗∗∗*p* < 0.0001.

Immune cluster B had a considerably higher ICB score than immune cluster A (Figure 4A). The difference in the composition ratio of key clinical features between the low and high ICB score groups was evaluated using chi-square analysis. The clinical heatmap (Figure 4B) highlighted the clinical features of patients in the TCGA cohort, and it was discovered that age, Breslow depth, Clerk level, AJCC stage, and T stage differed significantly between the low- and high-score groups. To reveal the differences in ICB scores with previously identified immune clusters, TCGA subtypes (Cancer Genome Atlas Network, 2015), and immune subtypes (Thorsson et al., 2018), we illustrated the distribution of patients in the TCGA-SKCM cohort with an alluvial diagram (Figure 4C). It was found that patients with immune-inflammatory subtypes were concentrated in the high ICB score group and that patients with low ICB scores had a higher survival rate. GSEA of melanoma samples was used to further investigate the features of the low and high ICB groups. The low ICB score was enriched in immune activation pathways such as immune response activation, alpha-beta T cell activation, and antigen receptor-mediated signaling, as shown in Figure 4D. Surprisingly, the high ICB score group was enriched in keratinocyte-related pathways, such as cornification, keratinization, cornified envelope, and desmosome pathways annotated by GO terms (Figure 4E). We then focused on the core biological processes involved in the anti-tumor immune response. We assessed a set of genes related to the specific biological processes identified by Mariathasan et al. (Mariathasan et al., 2018). In this study, the low IMS group showed high expression of stromal activation pathways such as EMT1, EMT3, and FGFRG3 associated genes. In the high-ICB group, CD8 effector and antigen presentation markers were expressed at higher levels (Figure 4F). In addition, cell cycle and DNA replication pathways were substantially expressed in those with a high ICB score. Spearman correlation analysis indicated that these signatures were significantly associated with the ICB score (Figure 4G). DNA damage repair systems are vital for maintaining genome integrity (O'Connor, 2015). Rosenberg et al. found that the alteration of DDR pathway-related genes was significantly associated with a higher tumor mutation burden and predicted the optimized anti-PD-1/PD-L1 immunotherapy efficiency (Arora et al., 2019) (Teo et al., 2018). Therefore, we evaluated the relationship between the DNA damage repair pathways and ICB scores. The ICB score showed a positive correlation with the enrichment score of the DNA damage repair pathway (Spearman’s R = 0.23, *p* < 0.001, Figure 4H). In addition, five (BER, FA, NER, HR, and NHEJ) of the six DNA damage repair signatures were significantly differentially enriched between the low and high ICB scores (Figures 4I,J).

### The Immune Landscape of the Low and High ICB Score Group

**FIGURE 5:**
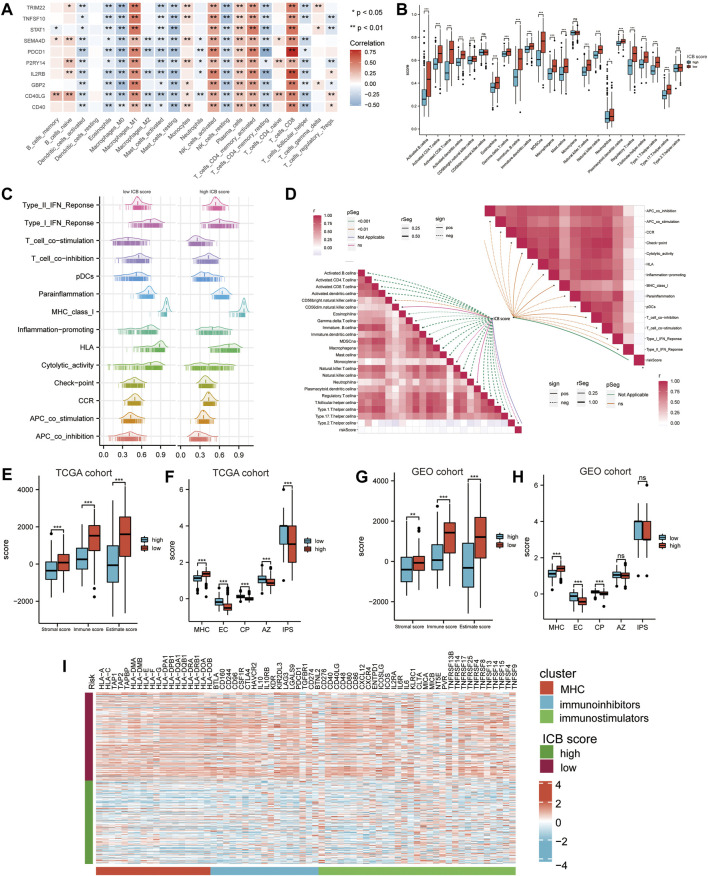
Correlation between the ICB score and potential immune characteristics in melanoma. **(A)** Correlations heatmap shows the association of immune infiltrating levels of TIICs estimated by CIBERSORT with ICB signature genes. ∗*p* < 0.05, ∗∗*p* < 0.01. **(B)** Differences in the immune infiltrating level of 22 TIICs between the low and high ICB score groups using the CIBERSORT method. **(C)** GSVA analyses revealing differences in immune function related pathways between the low and high ICB scores. **(D)** Correlation heatmap showing the correlation between the ICB score and the enrichment score of immune cells infiltrating and immune function related pathways. **(E)** Differences in the immune score, stromal score, and ESTIMATE score in TCGA-SKCM cohort. **(F)** Differences in IPS score, including MHC, EC, CP, AZ, and IPS score, between the low and high ICB score groups in TCGA-SKCM cohort. **(G)** Differences in the immune score, stromal score, and ESTIMATE score in GSE65904 cohort. **(H)** Differences in IPS score, including MHC, EC, CP, AZ, and IPS score between the low and high ICB score groups in GSE65904 cohort. **(I)** Heatmap showing the differences in the expression of immune molecules between the low and high ICB score groups in TGCA-SKCM cohort. Strips below the heatmap represented the types of immune molecules. Red, MHC molecule; blue, immune inhibitors; green, immune stimulators. ∗*p* < 0.05, ∗∗*p* < 0.01, ∗∗∗*p* < 0.001, ∗∗∗∗*p* < 0.0001.

We accessed the correlation between genes in ICB score and tumor infiltrating immune cells (TIICs) evaluated by “Cibersort’ methods. They showed positive correlations with Macrophages M1, NK cells, and CD8+ T cells, considered the primary effector cells and Antigen presentation cells in the anti-tumor immune response (Figure 5A). The ssGSEA results revealed that the low ICB score groups shared a markedly higher immune infiltration level in almost all types of TIICs. The enrichment scores of immune function-related pathways calculated using ssGSEA methods differed in the low and high ICB score groups (Figure 5B). The correlation heatmap revealed that the ICB score was positively correlated with the infiltration level of TIICs and both immune stimulation and inhibition pathways (Figures 5C,D). Moreover, the lower ICB score groups shared higher immune and stromal scores in TCGA-SKCM (Figure 5E), indicating more excellent immune cell and stromal cell infiltration. Charoentong et al. developed an immunophenoscore (IPS) that includes MHC (Antigen processing), CP (checkpoint), EC (effector cells), and (suppressor cells) to predict the efficiency of anti- CTLA-4 and anti-PD-1 antibodies therapy. We evaluated the differences in MHC, EC, CP, and average Z-score (AZ) between the low and high ICB score groups. In the TCGA-SKCM cohort, EC, CP, AZ score, and IPS were higher in the low ICB score group than in the high score group, while the high score groups shared a higher MHC score (Figure 5F). In the GSE65904 cohort, higher immune and stromal score (Figure 5G), higher EC and CP scores were represented in the low ICB score groups, whereas MHC scores were lower in the low ICB score groups, similar to the results in the TCGA cohort. However, in GSE65904, there was no significant variation in IPS scores between the low- and high-score groups (Figure 5H). The expression of immunomodulatory molecules was also examined in the high and low ICB groups. The results revealed that most immunomodulatory molecules were expressed at higher levels in the low-ICB score group than in the low-score group (Figure 5I).

### ICB Score Related Genomic Alterations in the TCGA-SKCM Cohort

**FIGURE 6:**
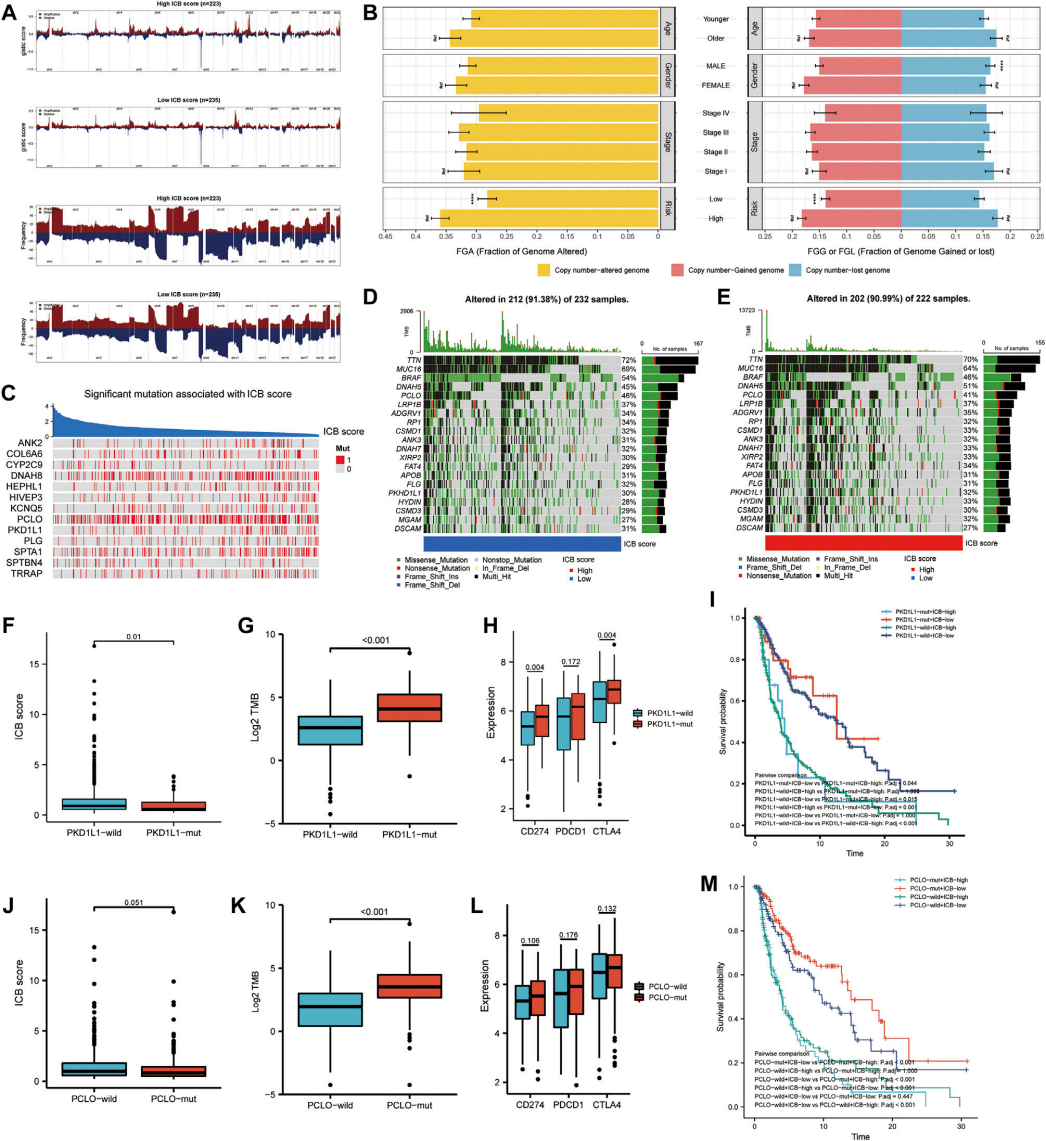
Correlation of genomic alterations with the ICB score in melanoma. **(A)** Comparison of the somatic copy number variations (SCNVs) between the high ICB score group and the low ICB score group in the TCGA-SKCM cohort. **(B)** Differences in fraction genome altered (FGA), fraction genome gain (FGG), and fraction genome loss (FGL) values in different ages, genders, TNM stage, and ICB score groups. ∗*p* < 0.05, ∗∗*p* < 0.01, ∗∗∗*p* < 0.001, ∗∗∗∗*p* < 0.0001. **(C)** Heatmap showing mutations in the genes with the most significant correlation to ICB score in melanoma samples. Genes with P-values lower than 0.0001 were exhibited in the figure **(D–E)** Top 20 most frequently mutated genes in the low and high ICB score groups. **(F)** The difference in ICB score between PKD1L1-wild and PKD1L1-mut samples. **(G)** The difference in TMB between PKD1L1-wild and PKD1L1-mut samples. **(H)** Differences in classical immune checkpoints (PD-1, PD-L1, and CTLA-4) between PKD1L1-wild and PKD1L1-mut samples. **(I)** Survival analyses for subgroup patients stratified by ICB score and PKD1L1 mutation status using Kaplan-Meier curves. Adjusted P values of multiple hypothesis test results were calculated using the Bonferroni method. **(J)** Difference in ICB score between PKD1L1-wild and PKD1L1-mut samples **(K)** Difference in TMB between PKD1L1-wild and PKD1L1-mut samples. **(L)** Differences in classical immune checkpoints (PD-1, PD-L1, and CTLA-4) between PKD1L1-wild and PKD1L1-mut samples **(M)** Survival analyses for subgroup patients stratified by ICB score and PKD1L1 mutation status using Kaplan-Meier curves. Adjusted P values of multiple hypothesis test results were calculated using the Bonferroni method.

We further investigated the link between the ICB score and genomic changes (including CNV alternation and mutation). First, we investigated copy number gain/loss frequencies and GISTIC scores in the low and high ICB score groups. The high ICB score group had a higher GISTIC score and a more significant CNV gain/loss frequency than the low ICB score group (Figure 6A). Next, we explored how the various subtypes differed in terms of fraction genome gain (FGG), fraction genome loss (FGL), and fraction genome change (FGA). Men had a significantly higher FGL than women, despite no significant differences at different ages or AJCC stages. Furthermore, the FGA and FGG levels in the high ICB score group were significantly higher than those in the low ICB score group. According to the data, CNV alteration had a minor impact on SKCM’s low ICB score of SKCM (Figure 6B).

We then identified the ICB score-correlated gene mutations. Spearman’s correlation analysis showed that the mutation status of 13 genes was significantly correlated with the ICB score (Figure 6C). The cut-off value was set at *p* < 0.05. Among these 13 ICB-associated mutated genes, PCLO and PKD1L1 were the 20 most frequently mutated genes. In addition, we analyzed the differences in the distribution of somatic mutations between low and high m6Ascore in the TCGA-SKCM cohort using the “maftools” package. MUC16, BRAF, and PCLO had a higher mutation frequency in the low ICB score group, while the mutation frequency of DNAH5, FAT4, HYDIN, and MGAM in the high ICB score group was higher than that in the low score group (Figures 6D,E). We further evaluated whether PKD1L1 and PCLO mutations are potential intrinsic immune escape mechanisms in the low- and high-ICB groups. The results showed that the ICB score was significantly higher in PKC1L1-wild type patients than in PKD1L1-mut patients (*p* = 0.01, Figure 6F). TMB (*p* < 0.001, Figure 6G) and checkpoint expression (Figure 6H), including PD-L1(*p* = 0.004) and CTLA4 (*p* = 0.004), were significantly overexpressed in PKD1L1-mut patients. The survival curve suggested that PKD1L1 mutation did not weaken the prognostic ability of the ICB score (Figure 6I). In addition, the ICB score was elevated in PCLO-wild-type patients (Figure 6J). The TMB of PCLO-mut patients was significantly higher than that of PCLO-wild-type patients (Figure 6K). The intermediate value of immune checkpoint molecules in PCLO-mut was higher than that in PCLO-wild, although the results were not statistically significant (Figure 6L). In both the PCLO-wild and PCLO-mut groups, patients with lower ICB scores had prolonged OS, consistent with the PKD1L1 mutation (Figure 6M). Our results on genomic alterations shed light on the crucial role of PCLO and PKD1L1 mutations in shaping the SKCM immune microenvironment. However, the specific mechanisms of the correlation between PCLO, PKD1L1 mutation, and checkpoint expression need to be explored in further studies.

### ICB Score Predicts Response to Immune Checkpoints Blockade Therapy

**FIGURE 7:**
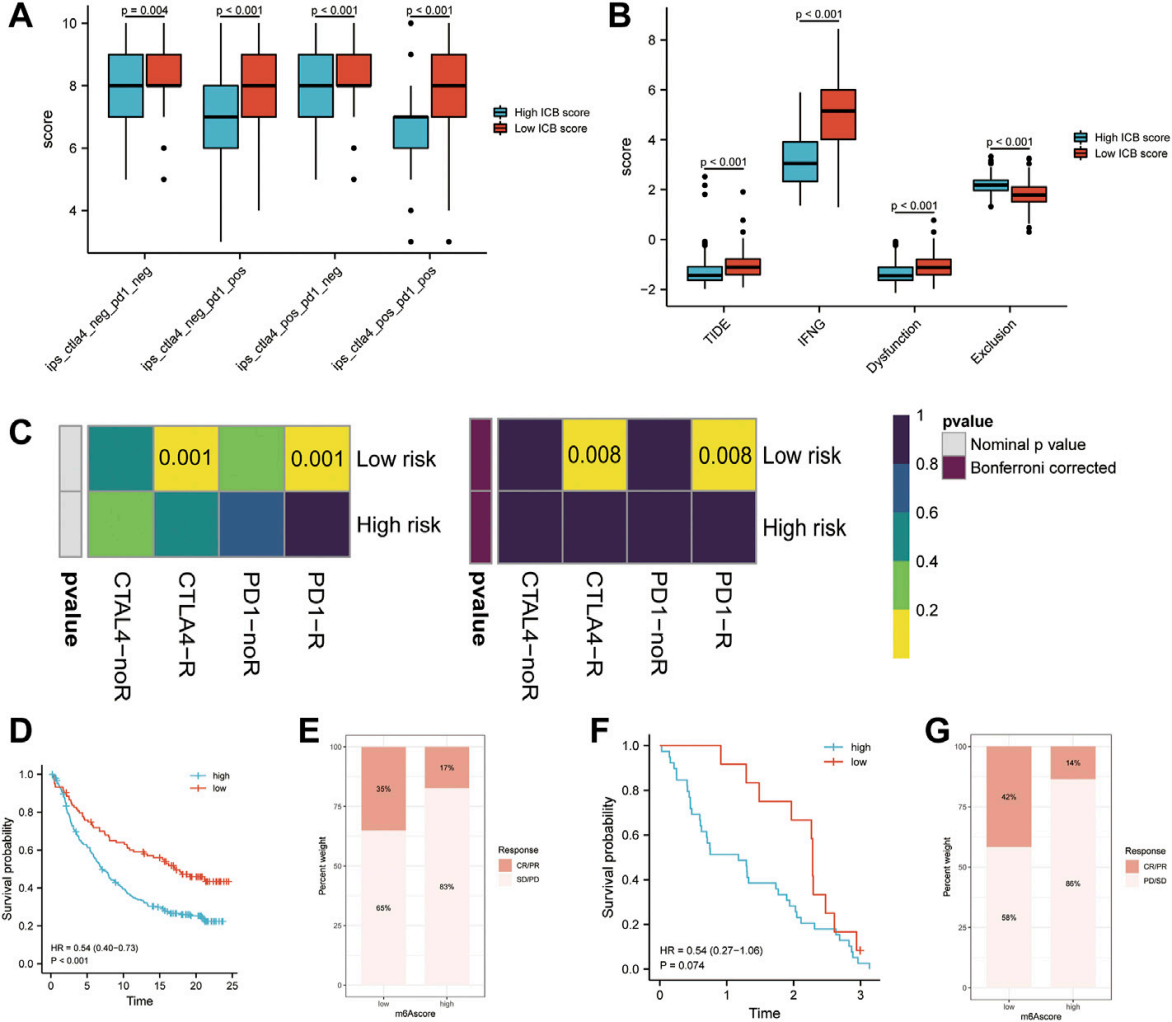
Prediction of the efficiency of immune checkpoint blockade treatment. **(A)** Differences in Immunophenoscore (IPS) between the low and high ICB score groups. **(B)** Differences in Tumor Immune Dysfunction and Exclusion (TIDE) between the low and high ICB score groups. **(C)** The Tumor Immune Dysfunction and Exclusion (TIDE) algorithm showed that the high ICB score group responded to PD-1/PD-L1 inhibitor treatment, while the low IMS group did not respond to immune checkpoint inhibitor treatment. **(D)** Survival analyses for patients with low and high ICB scores using the Kaplan-Meier method in the anti-PD-L1 cohort (IMvigor210). **(E)** The proportion of patients responding to PD-L1 blockade immunotherapy in the low or high ICB score groups in the anti-PD-L1 cohort (IMvigor210). **(F)** Survival analyses for patients with low and high ICB scores using the Kaplan-Meier method in the anti-PD-1 cohort (GSE91061). **(G)** The proportion of patients responding to PD-1 blockade immunotherapy in the low or high ICB score groups in the anti-PD-1 cohort (GSE91061).

As checkpoint blockers are only effective in a small percentage of patients, identifying predictive signs and mechanisms of immunotherapy resistance is a hot topic of study. In addition to well-known TIILs, TMB(49), clonal neoantigens (Robbins et al., 2013), and PD-L1 expression, newly identified signatures or algorithms, such as immunophenocscore (IPS) (Charoentong et al., 2017) and tumor immune dysfunction and exclusion (TIDE) (Roh et al., 2017; Fu et al., 2020)^,^ (Jiang et al., 2018), are widely used to predict response to immunotherapy. For this reason, we calculated the IPS of patients in the TCGA-SKCM cohort and found that the IPS of anti-PD-1 immune checkpoint therapy and anti-CTLA-4 immune checkpoint therapy (*p* < 0.001, Figure 7A) was significantly higher in the low m6Ascore group, which indicates that patients with low m6Ascore may get better efficiency from these two types of immunotherapies. The TIDE score and the IFNG genes and dysfunction signature scores were significantly higher in the low ICB score group, while the score of the immune exclusion signature was lower in the low ICB score group (Figure 7B). The TIDE algorithm was used to predict the efficiency of low and high ICB scores for immune checkpoint blockade (anti-PD-1/anti-PD-L1 and anti-CTLA4) treatment. The results suggested that patients with low ICB scores were more likely to respond to anti-CTLA4 or anti-PD-1/anti-PD-L1 treatment in SKCM, whereas those with high ICB scores were not. In addition to the TIDE algorithm, we also employed subclass mapping to compare the expression profiles of these two groups, which we defined with another published dataset encompassing 47 patients with melanoma who showed favorable responses to immunotherapies. We found that patients with a low ICB score were more likely to respond to anti–PD-1/CTLA-4 treatment. Both treatments had Bonferroni-corrected P-values of 0.008 (Figure 7C). We then evaluated whether the ICB score had a good performance in predicting patients’ response to ICB therapy in the immunotherapy cohort, given the close link between the ICB score and TIICs, immune function pathways, immune checkpoint expression, and immunotherapy predictors. Patients with a low ICB score had more substantial therapeutic improvements and longer life in both the anti-PD-L1 (IMvigor210) and anti-PD-1 (GSE91061) cohorts (IMvigor210, HR:0.54 (0.40–0.73), Figures 7D,E; GSE91061, HR:0.54 (0.27–1.06), Figures 7F,G). A previous study verified the considerable therapeutic benefits and clinical response to anti-PD-1/L1 immunotherapy in patients with low ICB scores compared with those with high ICB scores.

### Construction of Nomogram

**FIGURE 8:**
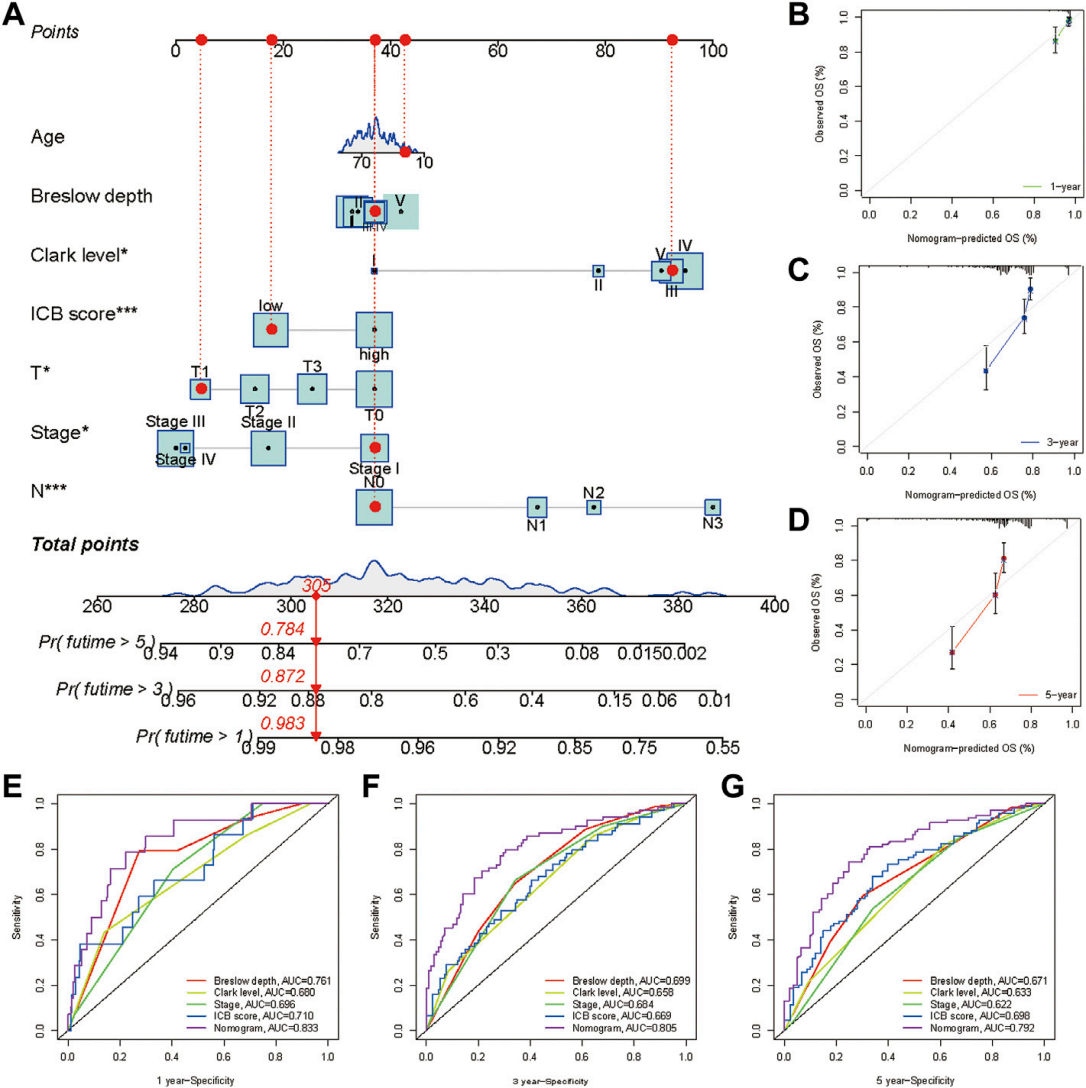
Construction and validation of a nomogram. **(A)** Nomogram integrated the ICB score, age, Breslow depth, Clerk level, T stage, N stage, and TNM stage. Breslow depth:level Ⅰ represents <0.75 mm; Level Ⅱ represents 0.75–1.50 mm; Level Ⅲ represents 1.51–3.00 mm; Level Ⅳ represents 3.01–4.50 mm; Level Ⅴ represents >4.50 mm**(B–D)** Calibration curve for predicting OS at 1, 3, and 5 years **(E–G)** Time dependent ROC curves of the nomogram, the ICB score, Breslow depth, Clerk level, and TNM stage at 1, 3, and 5 years.

A nomogram, including the ICB score and primary clinical parameters, was created to provide doctors with a quantitative method to predict the prognosis of patients with melanoma (Figure 8A). TNM stage, Breslow depth, Clerk level, tumor size, and N stage were the clinical parameters included in the nomogram. A point scale was used to specify the points for these variables in the nomogram. A straight line was drawn upward to calculate the points for the variables, and the total points allocated to each parameter ranged from 0 to 100. The points of the variables were accumulated and recorded as total points. To measure the risk of melanoma patient survival at 1, 3, and 5 years, a vertical line was drawn from the axis at the total point, straight down to the outcome axis. In addition, we found that the bias-corrected line of the calibration plot was close to the ideal curve, a 45-degree line in the plot (Figures 8B–D). The results indicated that the forecasts and observations for 1, 3, and 5 years were in good agreement. We then measured the predictive ability of this nomogram by comparing it with Breslow depth, Clerk level, and TNM stage. The nomogram performance (AUC:0.833, 0.805, and 0.792 at 1, 3, and 5 years, respectively) was better than Breslow depth, Clerk level, TNM stage, and ICB score (Figures 8E–G). Overall, our data implied that the nomogram outperformed individual prognostic markers in predicting short- and long-term survival in patients with melanoma (Rizvi et al., 2015).

## Discussion

Biomarker development for clinical checkpoint inhibition is still in its early stages of development. In certain cases, PD-L1 expression and TMB are the only biomarkers that can be used. Recent research has found that PD-L1 expression measured by immunohistochemistry (IHC) and TMB measured by whole exome sequencing (WES) are not reliable predictors of ICB response in a variety of tumor types (McGrail, Pilié, Rashid, Voorwerk, Slagter, Kok, et al.) (Merino et al., 2020). Tumor-infiltrating lymphocytes, molecular subtypes, and T-cell receptor clonality are currently being investigated as predictive biomarkers for ICB in various cancer types (Balar et al., 2017) (Thorsson et al., 2018). Pre-therapy expression analyses on pre-existent immune features in responders and expression patterns of “Cold” versus “Hot” tumors based on prior immunotherapy experience could predict immunotherapy response, according to mounting evidence (Riaz et al., 2017) (Galon and Bruni, 2019) (Ochoa de Olza et al., 2020). Therefore, identifying genes significantly related to the efficacy of immunotherapy from the perspective of the whole transcriptome will provide new insights into the intrinsic heterogeneity of immunotherapeutic response or non-response tumors. WGCNA is a classic data reduction and unsupervised classification method, which has been employed in thousands of transcriptional data analyses (Langfelder and Horvath, 2008). There is a consensus that the expression pattern of the patient’s transcriptome before treatment predominantly affects the efficacy of immune checkpoint therapy. However, as its complex and dynamic nature, our understanding of the expression feature relating to the efficiency of immunotherapy remains incomplete (Chen and Mellman, 2017). For the first time, we applied the WGCNA method to mRNA sequencing data of patients receiving anti-PD-1 therapy to discern the hub gene modules directly associated with therapeutic response.

In this study, we identified three modules (yellow, grey60, and light-green) that were significantly correlated with immunotherapeutic response. Pathway enrichment analysis revealed that the three key modules were enriched in immune function-, ECM formation-, and ATP metabolism-related pathways. Extracellular matrix components secreted by cancer-associated fibroblasts (CAFs) and metabolic reprogramming mediated by the hypermetabolism of tumor cells have been demonstrated to be involved in tumor immune evasion (Hilmi et al., 2020) (Hanahan and Weinberg, 2011). The activation of stroma was shown in cancers with an immune-excluded phenotype, which had an abundance of immune cells, but the effector cells remained in the extracellular component and failed to infiltrate the tumor, reducing the efficacy of immunotherapy (Chen and Mellman, 2017). The unique glycolysis metabolism of tumor cells provides energy for their proliferation and growth, and consumes a large amount of nutrients in the tumor microenvironment. The activation and effector functions of immune cells require a large amount of energy. Therefore, there is intense metabolic competition and close metabolic regulation between immune and tumor cells, which is another crucial mechanism of tumor immune evasion (Monteran and Erez, 2019) (Chang et al., 2015) (Pavlova and Thompson, 2016). By adjusting the balance between tumor and immune metabolism and targeting the ECM formation signals in the TME, they may point to new ways to increase the response rate of tumor immunotherapy (Pico de Coaña et al., 2015) (Lv et al., 2021) (Topalian et al., 2016). The above results demonstrated that the key modules identified by WGCNA described the three pivotal biological processes involved in tumor immune escape. Thus, we selected these three modules as the key modules for conducting subsequent analyses. Based on these key modules, we identified 2 transcriptional subtypes. We termed these immune clusters A and B. These two subtypes exhibit distinct immune infiltration characteristics. Immune cluster A was distinguished by the abundance of infiltrating immune cells and enrichment of immune activation pathways. The immunological landscape in immune cluster B was in contrast to that in cluster A. A recent study found that the tumor microenvironment influences tumor growth and immunotherapeutic effectiveness (Galon and Bruni, 2019). The probability of an immune response and a good prognosis have been linked to the baseline numbers of tumor-infiltrating cells, such as CD4+/CD8+ T cells, NK cells, and macrophage M1 (63) (Zeng et al., 2020). Immune cluster A was also shown to be related to increased CD8+ T cell infiltration, mutation load, and PD-L1 expression, indicating that it may have predictive relevance for immunotherapy. Immune cluster A, unsurprisingly, had a significantly longer overall survival than immune cluster B.

In recent years, several gene signatures have been established to assess melanoma prognosis. These signatures encompassing numerous genes derived from RNA-seq or RT-PCR data had acceptable predictive capacities, but none could be used in clinical practice owing to the high-condition analysis setting or the absence of additional validations (The ICGC/TCGA Pan-Cancer Analysis of Whole Genomes Consortium, 2020) (Yu et al., 2020). Considering the individual heterogeneity of molecular features and the crucial role of the key modules we identified in characterizing the immune landscape, we constructed a signature to predict prognosis and immunotherapeutic efficiency. RNA-seq data from both TCGA and GEO cohorts indicated that the ICB score had a strong predictive ability. Our signature was an independent risk factor for melanoma prognosis, and the nomogram incorporating clinicopathologic features and the ICB score had an excellent performance in predicting melanoma survival outcomes.

A subsequent study investigated the association between the ICB score and biological processes, tumor microenvironment characteristics, and genomic alterations. Increasing data suggest that solid tumors can be classified as either immunological inflammation (hot tumors) or immune exclusion (cold tumors), based on the presence of tumor-infiltrating immune cells or stromal activation (Binnewies et al., 2018). Consistent with the theory that higher infiltration levels confer a more favorable prognosis, patients with low risk showed upregulated immune activation pathways and higher infiltration of classical antitumor immune cells, while stromal activation-related pathways were significantly upregulated in high-risk tumors. Tumor immune escape mechanisms are critical for the development and progression of malignancies. Extrinsic immune escape pathways are represented by MHCs, ILs, and interferons, whereas intrinsic immune escape mechanisms are characterized by immunological checkpoints (Schreiber et al., 2011). Thus, we explored the difference in the expression of MHC, co-stimulators, and co-inhibitors between the low and high ICB score groups and discovered that these immune-related molecules were all upregulated in the low ICB score groups, which suggested that patients with a low ICB score experienced more intrinsic immune escape. At the same time, they had a higher level of immune infiltration. The essential variables affecting intrinsic escape are tumor immunogenicity and checkpoint molecule expression. Blocking the PD-1/PD-L1 pathway has been found to provide a long-lasting response in several malignancies by targeting intrinsic escape, thereby inducing immune cells to attack tumors. The results suggest that patients with a low ICB score may benefit more from immune checkpoint therapy. Genomic alterations represented by TMB are strongly correlated with immune phenotypes (Hegde and Chen, 2020). We investigated the whole exome sequencing data of TCGA-SKCM samples grouped according to the ICB score to explore the difference in the genomic landscape between high and low ICB scores. According to our findings, patients with a low ICB score had a considerably more significant proportion of CNV increase than those with a higher ICB score. In addition, there were differences in the gene mutation status between the high and low ICB score groups. The ICB score we developed was linked to TME features and genomic alterations, suggesting that it might reflect the inherent heterogeneity of patients with melanoma.

To investigate the association between ICB score and immunotherapy, we employed the IPS and TIDE algorithms to predict the response to immune checkpoint inhibitor (anti-CTLA4 and anti-PD-1/anti-PD-L1) treatment in patients with high and low ICB scores. The TIDE findings revealed that, in melanoma, high ICB scores reacted to anti-PD-1/anti-PD-L1 therapy, but low ICB scores did not (Figure 6B). In addition, we used anti-PD-L1 (IMvigor210) and anti-PD-1 (GSE91061) cohorts to assess various immune effectiveness markers. According to our findings, patients with low ICB scores had a significantly higher CR/PR rate. In summary, the ICB score demonstrated a high degree of precision in predicting the efficacy of immune checkpoint inhibitor treatment.

In short, the ICB score could be used in clinical practice to evaluate intrinsic and extrinsic immune escape processes and their corresponding TME cell infiltration characterization within individual patients and determine tumor immune phenotypes and guide more effective clinical practice. We also found that the ICB score was associated with the clinicopathological characteristics of patients. Similarly, the ICB score may be used to predict patient survival as an independent prognostic biomarker. The ICB score may potentially be used to predict the success of the clinical response of patients to anti-PD-1/PD-L1 immunotherapy. Our findings reveal a unique biomarker that may be used to distinguish between distinct tumor immune phenotypes, predict patients’ clinical response to immunotherapy, and improve customized cancer immunotherapy in the future.

However, our study had certain limitations. First, the ICB score was calculated using only a few genes chosen using LASSO algorithms, which did not adequately reflect the heterogeneity of the whole genome despite increasing clinical availability. Second, categorizing the melanoma samples based on the ideal ICB score was not an optimal technique for all samples; however, this problem was mitigated because correlation analysis was utilized in our study. However, more effective approaches to determine the appropriate cutoff value may be required. Furthermore, the transcriptional subgroups and ICB scores were discovered using retrospective records; therefore, a prospective cohort of melanoma patients undergoing immunotherapy is needed to confirm our findings.

## Data Availability

Publicly available datasets were analyzed in this study. This data can be found here: The datasets generated and/or analysed during the current study are available in TCGA database (https://tcga-data.nci.nih.gov/tcga/) and GEO database (https://www.ncbi.nlm.nih.gov/).
