# Supplementary material for: Identification of Crucial Gene Modules Related to the Efficiency of Anti-PD-1/PD-L1 Therapy and Comprehensive Analyses of a Novel Signature Based on These Modules
Source: Front Genet. 2022 Jul 22;13:893380. doi: 10.3389/fgene.2022.893380 (PMC9354784; doi:10.3389/fgene.2022.893380)
Supplement: Supplementary file 1 [file Table1.DOCX]

**Supplementary table 1.** Characteristics of melanoma patients in TCGA database.

| Clinical characteristics (total) |  | counts | Percent |
| --- | --- | --- | --- |
| Age at diagnosis (years) |  | 58.2(15-90) |  |
| Gender (460) | Female | 175 | 38.0% |
|  | Male | 285 | 62.0% |
| Breslow_depth (355) | < 0.75 mm | 33 | 9.3% |
|  | 0.75-1.50 mm | 72 | 20.3% |
|  | 1.51-3.00 mm | 77 | 21.7% |
|  | 3.01-4.50 mm  > 4.50 mm | 47  126 | 13.2%  35.5% |
| Clark level (317)  Stage (424) | Level Ⅰ  Level Ⅱ  Level Ⅲ  Level Ⅳ  Level Ⅴ  Stage 0  Stage Ⅰ | 5  18  76  166  52  16  77 | 1.6%  5.7%  24.0%  52.4%  16.4%  3.8%  18.2% |
|  | Stage Ⅱ | 139 | 32.8% |
|  | Stage Ⅲ | 170 | 40.1% |
|  | Stage Ⅳ | 22 | 5.2% |
| Tumor status (389) | T0  T1 | 30  42 | 7.7%  10.8% |
|  | T2 | 76 | 19.5% |
|  | T3 | 89 | 22.9% |
|  | T4 | 152 | 39.1% |
| Distant metastasis (434) | Negative (M0) | 411 | 94.7% |
|  | Positive (M1) | 23 | 5.3% |
| lymph node staging (406) | N0  N1  N2 | 229  73  49 | 56.4%  18.0%  12.1% |
|  | N3 | 55 | 13.5% |
