# Supplementary figures and images for: Identification of Crucial Gene Modules Related to the Efficiency of Anti-PD-1/PD-L1 Therapy and Comprehensive Analyses of a Novel Signature Based on These Modules
Source: Front Genet. 2022 Jul 22;13:893380. doi: 10.3389/fgene.2022.893380 (PMC9354784; doi:10.3389/fgene.2022.893380)

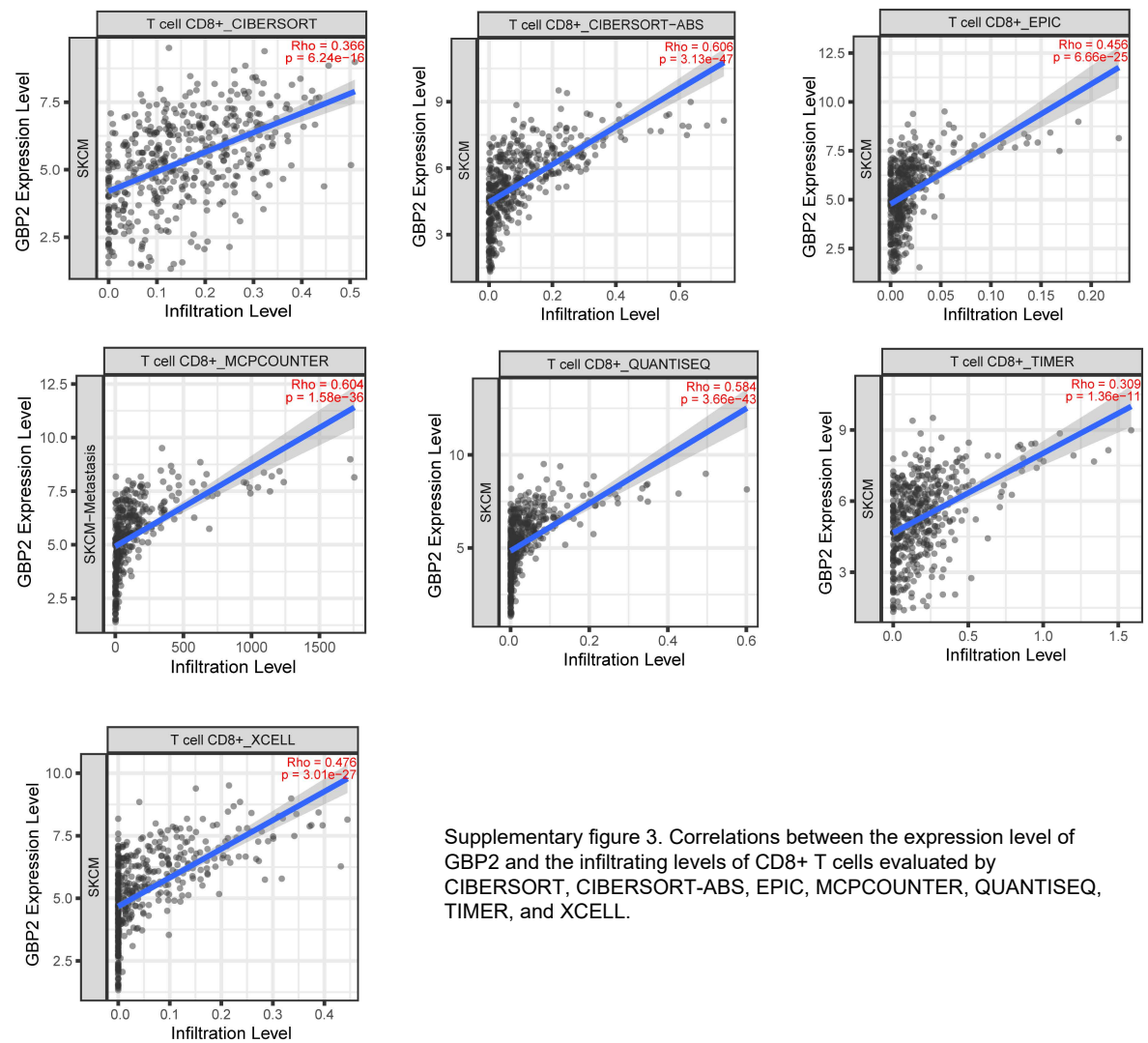

Supplement: Supplementary file 3 [file Image3.pdf]

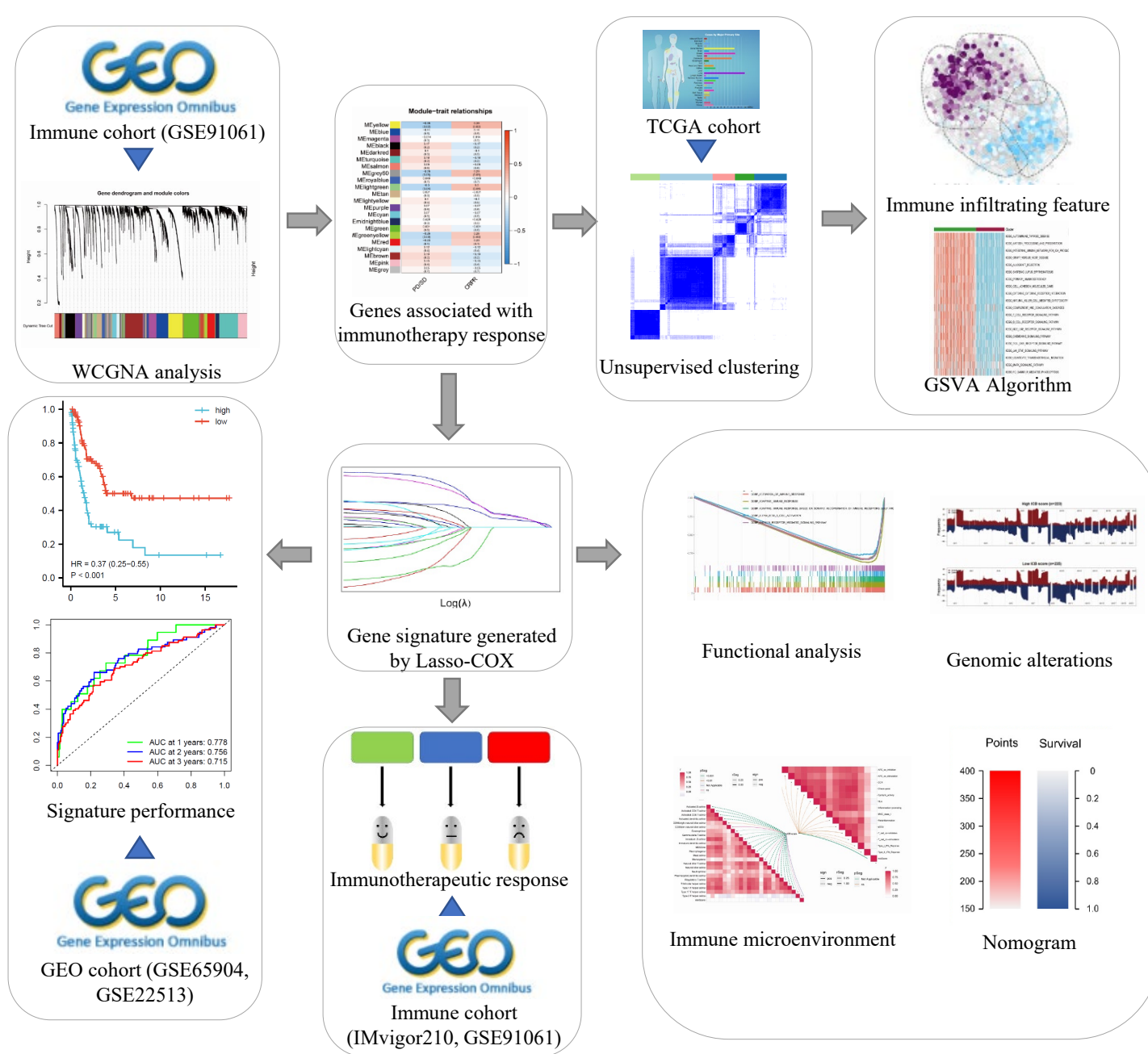

Supplementary figure 1: The workflow of this study.

Supplement: Supplementary file 4 [file Image1.pdf]
