# Supplementary material for: Identification of Crucial Gene Modules Related to the Efficiency of Anti-PD-1/PD-L1 Therapy and Comprehensive Analyses of a Novel Signature Based on These Modules
Source: Front Genet. 2022 Jul 22;13:893380. doi: 10.3389/fgene.2022.893380 (PMC9354784; doi:10.3389/fgene.2022.893380)
Supplement: Supplementary file 2 [file Image2.pdf]

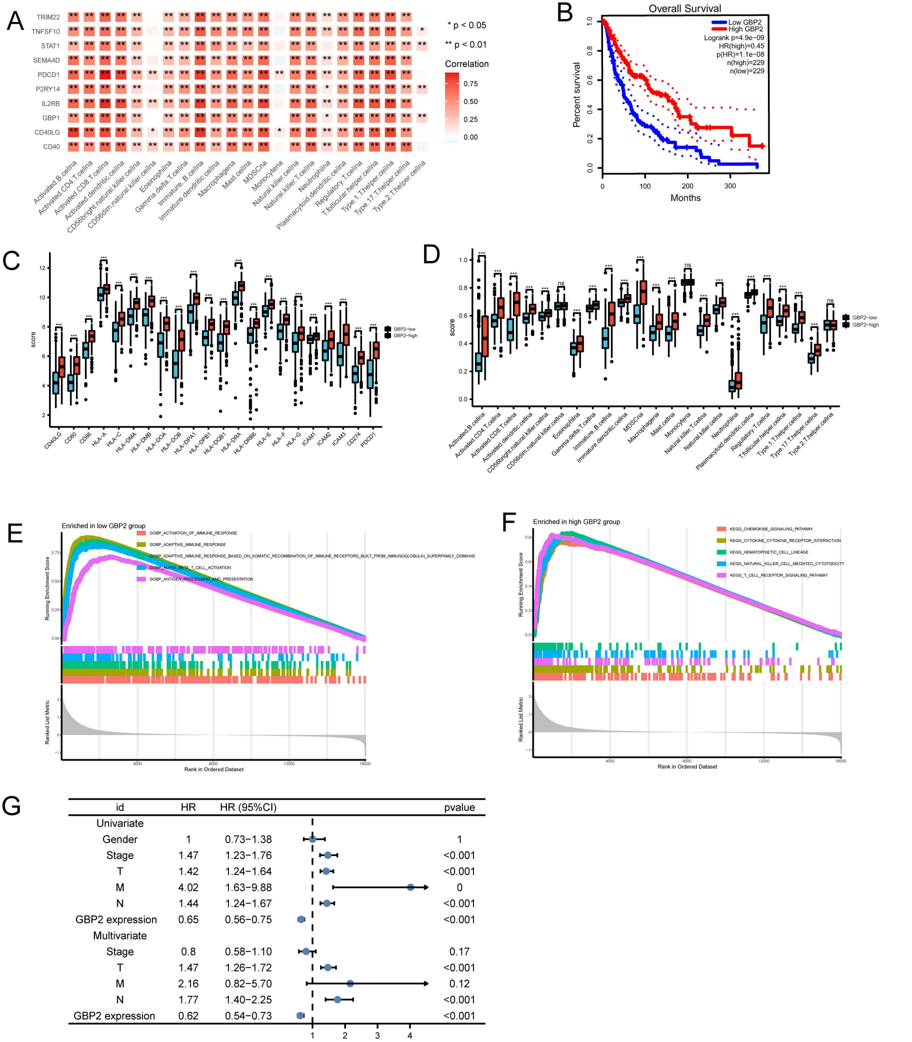

Supplementary figure 2: Correlations between immune characteristics and GBP2 and the prognostic role of GBP2 in melanoma. (a) The correlations between each type of tumor infiltrating cell with signature genes. Negative correlations were marked with blue and positive correlations with red. (*P < 0.05; **P < 0.01) (b) Survival analysis for patients with low or high GBP2 expression in TCGA-SKCM cohort. (c) Differences in the expression of MHC molecules, costimulatory molecules, and adhesion molecules between GBP2 high expression and low expression groups. (d) Differences in the abundance of each tumor infiltrating immune cell between GBP2 high expression and low expression groups. The upper and lower ends of the boxes represented interquartile ranges of values. The lines in the boxes represented median value, and black dots showed outliers. The asterisks represented the statistical p value. (*P < 0.05; **P < 0.01; ***P < 0.001) (e) The top 5 GO gene sets enriched in the high-expression group of GBP2. (f) The top 5 KEGG gene sets enriched in the high-expression group of GBP2. (g) Variables independently correlating to melanoma prognosis identified by the univariate and multivariate COX analysis.
